# Family medicine model in Turkey: a qualitative assessment from the perspectives of primary care workers

**DOI:** 10.1186/1471-2296-15-38

**Published:** 2014-02-27

**Authors:** Zeliha Asli Öcek, Meltem Çiçeklioğlu, Ummahan Yücel, Raziye Özdemir

**Affiliations:** 1 Ege University Faculty of Medicine, Department of Public Health, 35100 Izmir, Turkey; 2 Ege University Izmir Atatürk School of Health, Department of Midwifery, Izmir, Turkey; 3 Karabuk University School of Health, Department of Midwifery, Karabuk, Turkey

**Keywords:** Family physician, Family medicine, Primary care, Health care reform, Integration

## Abstract

**Background:**

A person-list-based family medicine model was introduced in Turkey during health care reforms. This study aimed to explore from primary care workers’ perspectives whether this model could achieve the cardinal functions of primary care and have an integrative position in the health care system.

**Methods:**

Four groups of primary care workers were included in this exploratory-descriptive study. The first two groups were family physicians (FP) (n = 51) and their ancillary personnel (n = 22). The other two groups were physicians (n = 44) and midwives/nurses (n = 11) working in community health centres. Participants were selected for maximum variation and 102 in-depth interviews and six focus groups were conducted using a semi-structured form.

**Results:**

Data analysis yielded five themes: accessibility, first-contact care, longitudinality, comprehensiveness, and coordination. Most participants stated that many people are not registered with any FP and that the majority of these belong to the most disadvantaged groups in society. FPs reported that 40-60% of patients on their lists have never received a service from them and the majority of those who use their services do not use FPs as the first point of contact. According to most participants, the list-based system improved the longitudinality of the relationship between FPs and patients. However, based on other statements, this improvement only applies to one quarter of the population. Whereas there was an improvement limited to a quantitative increase in services (immunisation, monitoring of pregnant women and infants) included in the performance-based contracting system, participants stated that services not among the performance targets, such as family planning, postpartum follow-ups, and chronic disease management, could be neglected. FPs admitted not being able to keep informed of services their patients had received at other health institutions. Half of the participants stated that the list-based system removed the possibility of evaluating the community as a whole.

**Conclusions:**

According to our findings, FPs have a limited role as the first point of contact and in giving longitudinal, comprehensive, and coordinated care. The family medicine model in Turkey is unable to provide a suitable structure to integrate health care services.

## Background

Fragmentation of services, lack of coherence, and domination by the paradigm of a disease-focused view that neglects the underlying causes of health and well-being are serious problems in many European health care systems [1,2]. Primary care is considered to be the key to overcoming these drawbacks of specialisation and building integrative health care systems that achieve better health and greater equity. Primary care’s four cardinal functions give it this central position: first contact (going to primary care first for each new need or problem), longitudinality (person-focused care over time), comprehensiveness (addressing all health-related needs in the population), and coordination (integrating care when patients have been seen elsewhere) [3-5]. These integrative functions of primary care are based on a person- and population-health focused view, which attempts to improve the equitable distribution of health and to link the biomedical, psychological, and social dimensions of health and well-being [5,6].

During the past two decades, primary care systems across Europe have been faced with extensive reforms such as decentralisation; provider deregulation; purchaser-provider split; implementation of market-like, contractual relationships and performance-based payment methods; introduction of commercial enterprises; and focus on consumer choice [7-14]. Nordic Europe and former socialist countries in particular have been subject to dramatic organisational changes, such as the introduction of person-list systems and a separation of the organisation of primary care and public health [9,10,12,13]. In former socialist countries, these reforms were mainly advocated and dictated by the World Bank [15,16]. Assessments of the effects of the reforms’ different components indicate an increased fragmentation in the health care sector and a loss of overall coherence in the organisation of primary care [9,10,12,17]. Turkey is among the countries that have undergone significant reforms in primary care. However, apart from the study of Kringos et al. [18], which was carried out when the reform was in the pilot phase in only two provinces (Eskisehir and Bolu), there has been limited work assessing the features of primary care in relation to its integrative structure.

### Primary care services in Turkey

Although dating back to the beginning of the 1990s, the real implementation phase of health reforms in Turkey started in 2003 under the Health Transformation Program (HTP) [19]. Although the HTP was initiated just after the Justice and Development Party took over the government, it is dictated and monitored by the World Bank [20-23]. Reforms covered a number of health policy areas in both the provision and financing of health services, but with a special emphasis on primary care services. A family medicine model within a performance-based contracting framework was first introduced as a pilot programme in 2005 and then extended to cover the whole country at the end of 2010 [23,24]. The introduction of this model altered the basic framework for the organisation of primary care, in which the district-oriented primary health care centre had been the established model.

The district-oriented model, called the “Socialisation of Health Services”, was constructed on a population-based structure with special emphasis on community participation and intersectoral action. The 1961 Law on Socialisation enforced the establishment of health centres serving a population of 5,000 to 10,000 in villages and 30,000 to 50,000 at the provincial level, staffed by teams comprising general practitioners, nurses, midwives, health officers, and environmental health technicians. The Socialisation of Health Services, which aimed to bring integrated primary care and public health services to even the remotest villages of the country, began to be implemented starting from the poorest region in 1963, and by 1983 it covered the whole country [24]. However, during the period of its implementation, the model could not find enough support from a number of successive governments and faced serious problems related to the policy-making process, such as poor management and supervision, abolition of the referral chain, a complete lack of infrastructure, unequal distribution of health staff, and insufficient funding for operating costs [24-27].

The introduction of the family medicine model separated the functions of health centres into two different organisations. Family health centres (FHCs) provide patient-specific preventive care services (immunisation and monitoring of pregnant women and infants) and diagnostic, curative, rehabilitative, and counselling services at the primary care level, whereas community health centres (CHCs) are responsible for activities at the community level, such as the collection of statistics, control of communicable diseases, environmental and occupational health services, health promotion and education services, and school health services [24]. Family physicians (FPs) are general practitioners and family medicine specialists providing primary care to the people on their lists [24]. FPs function with a midwife, a nurse, or an emergency medicine technician, who collectively can be conceived as a single family health unit called “family health workers” (FHWs) [23]. Thus, midwives, who used to play a key role in the community-based activities of health centres, have become FP assistants. In the introductory phase of the model each FP was assigned a population according to his/her location in the provinces, but after 6 months, patients were able to change their FP [24]. On average, 3,500 patients are registered to each FP, but the number of registered patients per FP can be as high as 4,500 [23].

FPs and FHWs are contracted for a period of 2 years, and payment is made on the basis of capitation adjusted by the socioeconomic development level of the region in which they work [23]. In addition, FPs receive additional payments to cover operational costs and laboratories. According to the performance aspect of the contracting scheme, failure to meet performance targets can result in payment cuts (up to 20% of their basic salary) and also contract termination for both FPs and FHWs. The salary deduction system is focused on three indicators: (1) the immunisation coverage rate of registered children; (2) monitoring of registered pregnant women with a minimum of four antenatal care visits according to the schedule; and (3) follow-up visits of registered infants. Compliance with governance and performance targets is assessed at least once every 6 months through a facility visit by CHC staff. A mandatory referral system from primary care to hospitals was initially included in the performance-based payment scheme. However, the government decided to abolish the referral system following 3 months of experience and now patients are free to enter the health care system at whatever point they choose and to use hospitals’ ambulatory outpatient services without needing a referral [23,24].

### Aim

This article’s study is part of a comprehensive project on the family medicine model in Turkey. The purpose of this study was to explore from primary care workers’ perspectives whether the family medicine model can achieve primary care’s cardinal functions (first contact, longitudinality, comprehensiveness, and coordination) and thus to understand whether the model has an integrative position in the Turkish health care system. Another part of this project investigated the impact of the introduction of the family medicine model on primary care workers and will be presented elsewhere.

## Methods

### Study design and participants

An exploratory descriptive design was used to investigate multi-dimensional and context-related features of primary care services. Four groups of primary care workers were included in the study: 1) FPs; 2) FHWs; 3) CHC physicians; and 4) midwives and nurses working in CHCs. Maximum variation sampling was used to reflect the different socio-demographic characteristics of the primary care workers, such as age, sex, years in the profession and in primary care, and the presence of any PhD degree or specialty. To ensure a diversity of provinces, the nomenclature of territorial units for statistics of Turkey was considered and for each of the 12 subregions which make up level one, participants from at least one province were invited to participate in the study.

**Figure 1:**
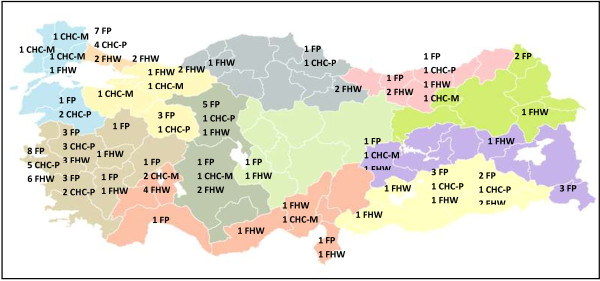
**The geographical distribution of the participants.** FP: Family Physician (n= 51), FHW: Family Health Worker (n= 44), CHC-P: Community Health Center Physician (n= 22), CHC-M: Community Health Center Midwife/Nurse (n= 11).

Participants were initially telephoned or visited to explain the aim of the research and structure of the interview, to obtain their consent for tape recording, and to set an appointment for an interview. Eight CHC physicians and two FPs refused to join the study. Recruitment continued while new information was emerging and ceased when saturation was achieved. The final study population consisted of 128 primary care workers from 38 different provinces (Figure 1). For practical purposes, a greater proportion of the participants were working in the west of Turkey.

Ethics committee approval was obtained from the Ege University Faculty of Medicine Research Ethics Committee (No. 12-5.1/17).

### Data collection

Data were collected using 102 in-depth and six focus-group interviews conducted between February and July 2013. A semi-structured interview form, based on the operational definitions of the four cardinal features of primary care, was developed during a workshop with the participation of all members of the project team. A pilot study involving two FPs, one CHC physician, two FHWs, and one CHC midwife was performed to improve the understandability and content validity of the form. Table 1 shows the final form. Interviews were conducted by the project team in environments suitable to ensure confidentiality and tape recording. In-depth interviews lasted 45-60 minutes, whereas focus group interviews were 60-90 minutes.

**Table 1:** Questions included in the semi-structured interview form

	
1.	Could you please provide some brief background information about yourself and which institutions you have previously worked for?
2.	What are the most fundamental differences between the family medicine model and the health centre system?
3.	Could you please compare the patient-list system with geographically-based organisation as a model of organisation?
4.	Could you please evaluate the family medicine model with regards to how easy it is for the community to access services?
5.	In your opinion, does the family medicine system fulfil the primary care function of first point of contact?
6.	Could you please evaluate the services provided in the family medicine system, such as the treatment of chronic disease, monitoring of pregnant women, etc?
7.	To what extent are you able to keep informed of the services your patients receive at other institutions from specialists and the results of their treatments?
8.	Are you able to carry out teamwork with other health staff employed in primary care settings?

### Data analysis

Each focus group was transcribed verbatim. First, two of the authors read the physicians’ interviews, while the other two authors (academics in the school of midwifery) read the interviews with FHWs and CHC midwives/nurses. All transcripts were then read together to see similarities and differences between the sets of answers. Researchers familiarised themselves with the raw data then discussed the conceptual framework of themes. After coding in Word files relevant words and sentences from each participant, these codes were combined in another Word file and sorted to visualise grouping of themes without losing the link with the original data. This was also done to develop a classification of themes. All the themes and theme categories were checked to see whether new categories or themes were needed. Themes were grouped for reporting and each theme was illustrated by direct quotations from participants.

## Results

Demographic characteristics and professional experience of the 128 primary care workers who were interviewed in this study are presented in Table 2. Participants’ views on the family medicine model were gathered in relation to five topics. The first topic, accessibility, is a structural element of primary health care. The other topics are the cardinal functions of primary care.

**Table 2:** Overview of demographic characteristics and experiences of the partcipants

**Characteristics**		**Family physician**	**Community health center physician**	**Family health worker**	**Community health center midwife/nurse**
**Sex**	Female	18	7	40	11
	Male	33	15	4	-
**Age**	≤ 29 years	4	2	22	2
	30-39 years	12	2	18	8
	40-49 years	28	10	4	1
	≥ 50 years	7	8	-	-
**Statistical region (Level 1)**	Istanbul	7	4	2	-
	West Marmara	1	2	1	3
	Aegean	16	10	11	-
	East Marmara	3	1	5	2
	West Anatolia	6	1	3	1
	Mediterranean	3	-	7	3
	Central Anatolia	1	-	1	-
	West Black Sea	1	1	3	-
	East Black Sea	2	1	3	1
	Northeast Anatolia	2	-	1	-
	Central Anatolia	4	-	2	1
	Southeast Anatolia	5	2	5	-
**Years in profession**	< 5 years	3	2	10	1
	5-14 years	8	2	18	4
	≥ 15 years	39	18	16	6
**Experince in family physician model**	≤ 3 years	38	-	36	-
	> 3 years	13	-	4	-
**Experience in health centre model**	Yes	41	20	31	10
	No	10	2	13	1
**Presence of any PhD degree or specialty**	No	40	20	44	11
	Family medicine	9	-	-	-
	Public health	2	2	-	-
**Total**		51	22	44	11

### Accessibility

#### ***Proportion of the population registered with family physicians***

Three out of five physicians and almost all FHWs stated that there were people who were not registered with any physician. They thought that the family medicine model ignored this segment of the population. The main reasons for the existence of this unregistered population were said to be a lack of field work and non-implementation of the referral system. The highest proportion of people outside the system was reported in areas with constant inward and outward migration. In addition, another group who remain outside the system are those who have not been recorded in the state’s census records (mostly children).

Two FPs were appointed to a region near us. They came without any patients on their lists. After one month, both of them had 3,000 patients, and there was no reduction in our patient numbers. This is because there is a large segment of the population who aren’t registered anywhere. We are in one of the regions of Ankara with the highest inward migration. (FP, 48 years old)

Even in areas with the highest socio-economic conditions, there may be a group to whom services cannot be provided, such as gypsies. They spend three months of the year here and there is no mechanism to meet their need for vaccinations. (FP, 45 years old)

#### ***Accessibility of care for those who are registered with family physicians***

Most of the participants reported that patients registered with FPs have accessibility problems because of a lack of planning when areas were assigned to physicians. These participants stated that during the transition to the family medicine model, patients who were registered with a physician far from their home, or who subsequently changed their place of residence, were turned away by some FPs in their local areas on the grounds that the FPs’ lists were full. It was also stated that the family medicine model gives rise to accessibility problems in rural and remote areas.

More than half of the participants emphasised the intensity of migration-related mobility in Turkey, and stated that sections of the population who constantly migrate have accessibility problems because they cannot use services from FPs other than their own. Students, seasonal workers, those who work far from home, those staying as guests in somebody else’s home, and those who regularly spend part of the year in another region frequently cannot access services when needed.

There are seasonal agricultural workers. They migrate for six months. The FP they go to there adds them to their own list, and the patient leaves your list. And when they come back, we don’t accept them. I say to the patient “Don’t try it on, there is no guarantee that when you go there, you won’t get pregnant.” (FP, 48 years old)

In addition to not seeing patients who are not on their lists, FPs’ patient-registration selection practices cause problems of accessibility. Statements made by the participants showed that FPs choose not to register patients with whom they find it difficult to communicate because of factors such as language, cultural differences, the possibility of migration, and the presence of serious health problems. Although one section of the population was reported as having difficulties with access, participants reported that another section of the population makes extensive use of services. This was reported as causing the daily number of patients at clinics to be very high (between 40 and 120), consequently drastically shortening the amount of time spent with each patient.

My patients are of a certain cultural level, which I try not to lower. It may seem like discrimination, but I can communicate more comfortably. (FP, 33 years old)

A person comes to register with me. The first question I ask is “How many people are there in your family, how many children?” and I think about it. Is this person going to wear me out? Is there anyone elderly or bedridden at home? Is there anyone disabled? If so, I don’t accept the patient. (FP, 29 years old)

The working day, which starts at 8 o’clock in the morning, may not finish at 5 o’clock in the evening because of the clinic. I take a look and realize that I have examined 100 patients. The time I have devoted to each patient is not even 4 minutes. (FP, 45 years old)

### First-contact care

FPs reported that 40-60% of the patients on their lists have never received a service from them. In regions where access to other health care services is easy, this percentage may reach the eighties. Participants stated that the majority of patients who use FP services do not use FHCs as their first point of contact when accessing health care services. This was related to the fact that the referral system is not applied in practice, and to a lack of trust in general practitioners in society. On the other hand, participants stressed that there are geographical differences in patients’ use of FHCs as a first point of contact. Whereas the proportion of patients using their FP as their first point of contact is at most 30% among FPs whose patients are relatively wealthy and work in the city centre, in rural areas this proportion can reach 80%.

50% of your patients rarely come, 25% make you work really hard, and 25% never come. (FP, 29 years old)

The vast majority of most participants thought that under present conditions introducing the function of gate keeping to primary care would have extremely negative consequences. Participants expressed these thoughts with statements such as “it would be complete chaos” and “everything would collapse”. Fifteen FPs and three CHC physicians reported that the number of patients registered with each FP was very high (3,500-4,000) and pointed out that implementation of a referral system would likely result in a huge increase in workload.

I have 4,000 patients, of whom 1,500 at the most visit me regularly. If the referral system were to be implemented, they would all come. There would be never-ending queues at the door. (FP, 37 years old)

### Longitudinality

Three quarters of FPs reported that the list-based system enabled them to get to know their patients well, take responsibility for them, monitor them, and have good communication with them. However, this relationship was limited to only one segment of FPs’ patients. Almost all FPs and FHWs explained that it was impossible to ensure longitudinality of care in a situation where patients mainly came to request repeat prescriptions and tests recommended by specialists.

I examine maybe ten patients a month. This is because the patients who come have been examined by a specialist and want to continue taking the same medicine. We offer to examine them to see whether it is necessary for them to continue. But no, they come to us for a prescription insisting that they must have that particular medicine. (FP, 43 years old)

When women get pregnant, they first go to a gynecologist. There, they are told which tests they should have. Then, they go to their FP to get the tests done. (FHW, 27 years old)

Almost all participants stated that in addition to preventing longitudinality of care, being able to change physicians was used by patients to put pressure on FPs. It was explained that patients who could not get the medicine they wanted threatened to change FPs.

My patient numbers have decreased. This is because I don’t do everything that is requested. I don’t prescribe drugs such as antibiotics without doing an examination. (FP, 47 years old)

Twelve FPs, 10 CHC physicians, and seven FHWs stated that the list-based system had completely removed the longitudinality of the relationship between primary care and the community.

I have patients from every neighbourhood in the district. … The absence of an organisation based on geography is a big drawback. When there is a situation which needs monitoring in the area where I work, such as a dog bite or an communicable disease, we can’t do anything if the patient isn’t registered with us. (FP, 45 years old)

I don’t know where my patients live as a group. When we worked in the health centre, our patient lists were defined; at the end of the year we knew who our patients were. Knowing this enabled us to see the health indicators of the community, such as birth rate and infant mortality rate. (FP, 48 years old)

### Comprehensiveness

All participants reported that under the family medicine model the best-run services were immunisation and monitoring of pregnant women and infants. However, participants also frequently pointed out that because home visits could not be made it was not possible to identify pregnancies and infants amongst those who were not registered. The vast majority of participants also stated that if the system of penalties for not hitting performance targets had not been implemented the rates of monitoring would not be as high. Furthermore, participants also admitted that these penalties for not hitting targets led to false declarations being made.

The minister says that the vaccination rate is 100%. Of course it is 100%. If you don’t vaccinate a registered patient, your money is cut. The problem is with the vaccinations of unregistered babies. In the past, our midwives used to go out into the neighbourhood, ask the neighbours, and identify all those with babies one by one. Now, we don’t do this. (FP, 42 years old)

Sometimes, in order to avoid a penalty, I report that I have given a vaccination which I haven’t because I know that the family didn’t come when I called them, but they would come three days later within that follow-up period. (FHW, 27 years old)

Seven FPs, eight CHC physicians, and 10 FHWs reported that there had been significant problems with the quality of monitoring of pregnant women and infants. These problems were because of an extremely high workload; the performance targets system; a lack of knowledge, skill, and cooperation among FHWs and FPs; and delays in the supply of medicines and immunisations. Participants admitted that during routine monitoring education and counselling services could not be given.

I have never heard any of the FHWs giving information. Height, weight, heartbeat, that’s all. Because they don’t have time. They have to spend time with pregnant women and new mothers, but they can only see them for about two minutes. Because either another patient comes in, or the midwife’s telephone rings, or the FP calls her. While one patient is having a vaccination or check-up, the next one comes in. (CHC midwife, 29 years old)

The majority of participants reported that services not included in the performance targets system, such as family planning, monitoring of children and new mothers, chronic disease management, and the reporting of communicable diseases, had been neglected. In particular, they reported that there had been a significant decline in family planning services, and that counselling and the fitting of intrauterine devices had been virtually abandoned.

For example, in our area teenage pregnancy is very common, but because postpartum services are not included in the performance system, they are not often given. (FHW, 26 years old)

I go to audit an FHC; is there a gynecological examination table? Yes, and the staff and doctor are also certified to fit intra-uterine devices. How many have you fitted in the last six months? None. The state doesn’t then say “Why don’t you fit them, you have been trained, you have the equipment.” (CHC physician, 64 years old)

Thirteen FPs reported that compared with the era of health centres, there had been an improvement in the monitoring of chronic diseases. Whether they thought these services were run better now than in the past, or whether they thought they were completely insufficient, all participants pointed out some important problems. The most frequently reported problem was that an extremely high workload precludes time for patient education or monitoring chronic diseases. Another problem was that because the referral system is not implemented and as the public does not have enough trust in primary care physicians, FPs find themselves in the position of being a person who just writes official prescriptions for medicines prescribed to patients by specialists.

I have identified my chronic patients, but I am not able to monitor them. There are two state hospitals, a whole load of private centres and two private hospitals near here. They are not dependent on me. (FP, 46 years old)

We only reach patients when they come to have their blood pressure taken or if they come because of another chronic illness, and we ask about the medicines they take. But we don’t know how long they have been taking the medicines, or whether they have been monitored. As for those who don’t come to us, we have absolutely no idea. (FP, 43 years old)

In the past, we used to be able to gather ten or fifteen people together and say “Come and let us give you information about breast cancer.” Now, even conducting breast examinations on those who come with a complaint is difficult, let alone giving education about breast cancer. (FHW, 27 years old)

Twenty-nine FPs identified home visits as one of the areas where they had the most problems. These physicians explained that because of their workload, and especially because of the high numbers of patients coming to their clinics, home visits were not realistic; they thought that the services provided by a single physician with limited time and inadequate equipment would not be effective. In addition, they stressed that without a mechanism which takes into account social problems, these services would be unable to achieve their goals. All FPs who provided home visits complained that patients made extreme demands and expected all of their health problems to be solved at home. The main reasons for this situation were reported to be the fact that the Ministry of Health had left the job descriptions ambiguous and had told the public that these services meant that a doctor would come to their home whenever needed.

I have two pregnant fifteen year old patients, one of whom is unmarried and had a miscarriage while being beaten, and I couldn’t send in the police… There are so many things which we can’t do. (FP, 51 years old)

What they demand is a service which could only be provided at a hospital, which could be provided by a physiotherapist, a psychologist, and a social service worker, for example; I mean by a team. One person alone cannot do anything. (FP, 48 years old)

Participants’ statements reflect the fact that the majority of communicable diseases go unreported. According to FPs’ statements the most important reason for this is the heavy workload produced by the processes needed for reporting. CHC physicians, on the other hand, thought that in addition to FPs’ workload, they didn’t report because of a lack of education and a lack of interest. Furthermore, participants explained that the system did not motivate the reporting of communicable diseases and in fact punished those who reported these diseases.

Because it is a lot of extra work. If you suspect a communicable disease, it is compulsory for you to obtain information from the secondary health care provider and monitor the patient. (FP, 45 years old)

There has never been proper reporting of communicable diseases. Whether in a hospital, or in a primary health care setting, the person who is going to make the report, and the manager’s assistant responsible for this at the head office both think “If I report this, how is it going to come back on me?” (CHC physician, 49 years old)

### Coordination of care

Three quarters of FPs reported that the majority of their patients had not been able to keep informed of the services they had received at other health institutions or of the diagnoses made by specialists. Participants identified that the fact the referral system was not implemented was the biggest obstacle to coordination, and reported that they only became aware of their patients’ situation when patients came to get an official prescription for medicines recommended by a specialist. Five FPs explained that they were unable to make effective use of the online system that collates patient information. Only three FPs reported that they had been able to work in cooperation with some specialists. Participants explained that coordination could only be achieved when patients informed their FPs about the services they received from other sources, and that this depended on the patients’ level of awareness.

In order to access the tests that patients have had done elsewhere, there is a data system, but opening it is problematic; you can reach data, for example you can reach a patient’s tomography, but you cannot reach the tomography report. (FP, 48 years old)

When I make a referral, I write the diagnosis and treatment, but the vast majority of specialists do not get back to me. Despite the fact that I write “I think it’s such and such an illness, I’ve done these things, what do you say?”, they don’t come back to me. (FP, 42 years old)

Half of FPs and all FHWs believed that team work had disappeared with the introduction of the family medicine model. FPs explained that services which used to be provided by a team at the health centre now had to be provided by individual FPs working alone with FHWs. Although there were participants who reported that physicians now felt more responsibility for preventive services like immunisation, in which they had not shown interest in the past, FHWs stated that these services were provided mainly by themselves, whereas the FPs’ interest was limited to hitting performance targets. In addition, FHWs stated that their working environment did not allow for the possibility of planning coordinated service with other health care workers.

One group, composed of the more experienced participants, stressed that the loss of team work not only stemmed from the fact that some of the team members had left and numbers had reduced, but that basic criteria such as making decisions as a team, setting targets, mutual development, and feeling collective responsibility were now impossible to realise.

A team of two people has to take on the workload of a health centre by themselves. In relation to reporting communicable diseases and monitoring pregnant women and infants, being an army of two is difficult. When we came face to face with these jobs ourselves, we understood what an important job our friends such as medical secretaries and environmental health officers do. (FP, 48 years old)

Generally, vaccinations and follow-ups are done by FHWs. As they constantly have to work in the clinic, FPs cannot devote time to these services. FHWs provide preventive services and FPs provide treatment services. (CHC physician, 36 years old)

Even in the same FHC, the physicians may not share the information that there has been a case of measles at a certain school. It wasn’t like this in the health centre system; we would share information such as there is an outbreak of flu in that school, or in this school we have started to see viral infections with rashes, and we would decide what to do. (CHC physician, 43 years old)

Half of the participants stated that the list-based system removed the possibility of evaluating the community as a whole and taking into account patients’ socioeconomic characteristics and changing needs. They felt that the cessation of home visits by midwives resulted in a breakdown of links to the community, which especially prevented the planning and provision of preventive services directed towards the community. In addition, there were FPs and FHWs who stated that the services which they provide cannot achieve anything if not integrated with social services.

In the past we also used to evaluate people according to their socioeconomic situation; we would look at where their toilet and kitchen were, where they got their drinking water from, and where they disposed of their waste. All that has finished. I ask patients what the problem is, and we treat whatever illness they have, that’s all. (FP, 42 years old)

In January 2011, there was an outbreak of measles in Istanbul. After investigating each case, taking blood samples and giving vaccinations, we would inform the FPs. It was unbelievable, nobody was interested! Patients had come to the FHC, been examined and sent to hospital, and that’s where it ended. Nobody asked whether there were any other children around them, whether there were any others who were ill, or what would happen to them. (CHC physician, 52 years old)

In the era of health centres, when there was a case of Hepatitis A in a child, their school would be within the regional boundaries and we would go to take a water sample, audit the canteen and identify other cases; their home would be within the regional boundaries and we would go and take a water sample and give education. It would all be sorted out in one place. Now, the place where the child lives, the FP they are registered with and the school are all separate. (CHC physician, 45 years old)

The ministry has focused on two things, clinic services and vaccinations. For example, solvent abuse amongst the young has increased in our neighbourhood. They have psychological problems, but nobody sees them. (FP, 40 years old)

There were some physicians who explained that the division of primary care into two parts, individual and community, and the division of these between two institutions, the FHCs and the CHCs, has led to significant problems. Participants’ reported that the definitions of the duties of the FHCs and CHCs could be confused, and that there could be delays in services such as school immunisations, investigations of the sources of communicable diseases, and the provision of death certificates because nobody took responsibility for them. It was reported that the relationship between FHCs and CHCs was based on audits, and that the loss of a team approach had turned these audits into just a method of punishment.

The CHC tries to get involved from the outside. It cannot coordinate, there is no integration; when you try artificially to separate something which is already integrated, problems arise. An unnecessary duplication of management and bureaucracy has been created. (FP, 48 years old)

## Discussion

This study provided information on the extent to which a family medicine model constructed on a capitation and pay-for-performance reimbursement structure and on a movement from community to professional-based care could achieve the cardinal functions of primary care and play an integrative role in a health system. Although the generalisability of its findings can be considered an important limitation, this study’s qualitative approach provided comprehensive and in-depth understanding of the perceptions and experiences of primary care providers. However, it should be noted that our findings are based only on health care providers’ perspectives and not that of the general public. Also, despite aiming for maximal diversity in our sampling approach, our study population may not have covered the full range of perceptions and experiences of all primary care providers in Turkey.

Accessibility is a structural element required for achieving the first-contact care function of primary care [2]. Two articles on Turkish health care reforms claim that the socioeconomic and geographic inequalities in access to care have been reduced dramatically since 2002 [28,29]. However, our findings indicate a different trend. A significant number of our participants emphasised that a non-negligible proportion of the population is still not registered with an FP. Moreover, the majority of those who are not registered belong to the most disadvantaged segments of the population, i.e., those most in need of primary care. Other than this, being registered with an FP does not guarantee access to primary care services. People can be turned away by FPs when they want to change their FPs to ones nearby or when they require health services while living in temporary places. According to our participants’ statements, the reason for this is related to FPs’ discriminatory patient selection practices and the fact that most FPs have lists of more than 4,000 patients. However, studies performed before the introduction of the family medicine model indicated that health centres played an absolutely critical role in meeting the heath care needs of population subgroups such as migrants, the poor, the unemployed, and the uninsured [30-34]. Another aspect of the access problem was expressed by our participants as the fact that the person-list-based system is not an appropriate organisational model for rural areas and dispersed settlements. This is in agreement with the assessments reported for Eskisehir, one of the pilot cities, indicating that rural areas were most affected by the drawbacks of the introduction of the new model [35].

According to the statements reported in this study, whereas the most disadvantaged subgroup of the population has limited access to primary care services, another group overuse and misuse FP services. Data from the Ministry of Health confirm the explosion in demand for services. The volume of primary care services (number of visits), which was 74.8 million in 2002, reached 244.3 million in 2011, while the number of primary care physicians increased from 17,800 to 22,073 in the same period. This means that the number of physicians went up by a factor of 1.2, while the number of visits increased by a factor of 3.2 [36]. When considering this contrast in the use of primary care services, we can claim that the Inverse Care Law, stated by Hart [37] as the principle that the availability of medical services tends to vary inversely with the need of the population served, is completely operative in Turkey. Rechel’s study presents a similar picture of the health care services in Bulgaria (where almost the same primary care reform process has been performed) and reports cultural, geographical, and financial barriers to children’s access to services, associated with poverty, poor education, and discrimination [12].

Kringos et al. [18], who assessed the family medicine model in two pilot provinces, Bolu and Eskisehir, reported that FPs had a position as first-contact care, especially for the health problems of women and children. However, the patient survey of the authors was composed of patients who visited FHCs. Considering that according to our participants almost half of the population do not use FPs’ services at all, this finding has the possibility of selection bias. According to our findings, FPs can play a first-contact role only for a limited segment of the population. Starfield and Boerma each stated that the first-contact function of primary care depended on the application of gatekeeping [2,38]. In agreement with this statement, the FPs in our study explained that the reason why they cannot function as a primary care provider is because patients can without restrictions access other levels of care. This situation does not leave FPs with a role other than giving repeat prescriptions and tests ordered by specialists. On the other hand, as Boerma (2003) noted, the FPs who participated in our study reported that the first-contact function may vary according to the socioeconomic features of the area and population served. FPs have a stronger first-contact position in rural areas, especially for poor and undereducated people.

Although participants pointed out the problems caused by the lack of gatekeeping mechanisms, almost all of them stated that under the current circumstances, introduction of a gatekeeping system could result in chaos. Experiences of other countries reveal drawbacks of gatekeeping mechanisms planned as part of health care reforms [12,39]. For instance, in 2001, Norway introduced a list-based system and a new payment scheme mainly based on capitation. The general practitioners who participated in a study conducted after the introduction of the new system admitted that they felt very uncomfortable when saying no to the referral demands of their patients in face to face relationships. The authors of this study concluded that the current economic incentives and increased patient autonomy do not combine well with making rationing decisions in primary care [39]. In Bulgaria, after the restriction of the number of referrals an FP could issue each month to specialists, many parents sought and paid for specialist paediatric services privately or relied on self-treatment; because of this, problems in children aged 0-14 years were exempted from the ‘regulatory standard’ [12]. Also, a World Bank report on the family medicine model of Turkey noted that the mandatory referral system which was initially included in the performance-based payment scheme of FPs had to be removed because it had created severe bottlenecks in the system and placed a high burden on FPs [23].

In the interviews the most positive statements about the family medicine model focused on the improvement in the longitudinality of the relationship between patients and providers. However, based on participants’ other statements it can be estimated that this improvement only applies to one quarter of the population. According to Starfield [2], central to the measurement of longitudinality is the idea that individuals should be able to identify their source of primary care and use this source for all health problems at the primary level. Furthermore, the primary care provider should be able to identify his/her eligible population through records on social characteristics, occupational and environmental exposures, housing conditions, and other factors [2]. However, our participants stated that there are still a lot of people in Turkey who do not know the FPs to whom they are assigned and that primary care is mainly used for the prescription of medicines and tests. Moreover, there is an absence of records required to assess the overall health of the patients. Also, in the study by Kringos et al. [18], patients were generally not convinced that the FPs were aware of their personal situation or the details of their medical history. Patient choice of provider is expected to improve the efficiency, quality, and responsiveness of the health system through the threat of exit [40,41].

On the other hand, according to studies on the determinants of the choice of primary care physicians, patients pay attention to a number of factors such as convenience, appearance of the office, and recommendations of friends or family, which may not accurately reflect the efficiency and quality of the services [42-44]. These factors are in line with the primary care providers’ statements in this study. However, according to our participants, the most important determinant of FP choice was the level of fulfilment of patient requests. Thus, the threat of exit does not result in an improvement in efficiency and quality in primary care in Turkey; on the contrary, it forces physicians to fulfil all the requests of patients without questioning their necessity, which also hampers the longitudinality of primary care.

There appeared to be general agreement among the participants that the best-run services provided by FHCs are the ones that are included in the performance-based contracting system as negative incentives, i.e., immunisation and monitoring of pregnant women and infants. Atun et al. also reported an increase in the percentage of FPs providing antenatal care and immunisation services on a daily basis [29]. However, the problems pointed out in the interviews raise questions regarding the ratio of pregnant women and infants not covered by the system, as well as the quality of the monitoring. Also, the World Bank report [23] referred to above admitted that the performance-based contracting scheme in Turkey started out with a mostly “pay for quantity” approach and does not incentivise the clinical process dimension in quality of care. The lack of quality indicators is not the only problem with the system. A more serious problem is indicated by the fact that the threat of performance points that result in salary deductions may bring about the misreporting of data. The measles outbreak that Turkey witnessed the last months of 2012 supports this concern about inaccurate data. Although the immunisation rates against measles for 2006-2011 were reported to be between 96% and 98% by the Ministry of Health [19], 43% of the confirmed cases aged 1-4 years had not been vaccinated [45].

Primary care providers’ comments support the claim that FPs have a low level of involvement in the provision of services that are not included in the performance indicators, such as family planning, postpartum follow-ups, and chronic disease management. This picture is completely in agreement with Starfield, who noted that the result of performance-based payment can only be an increase in measuring the measureable and physicians will do what they are paid to do [46]. According to her, performance-based payment has provided a mechanism for paying primary care physicians what they are worth, but there is no evidence that what has been valued is the most valuable in terms of health [46]. Similarly, a review including studies on payment-for-performance schemes implemented in the United Kingdom found evidence for only modest improvements in care, whereas the impacts on costs, professional behaviour, and patient experiences were uncertain [47]. In addition to giving priority to performance criteria, the loss of team work and the high daily workload—noticed also by Kringos et al. [18]—reduces consultation time, adversely affects the provider–patient relationship, and hinders FHCs’ provision of health promotion, preventive services, and education in Turkey. This change in the service profile is closely related to health care consumers’ demands. A study performed in Ankara in 2006, i.e., before the implementation of the family medicine model, reported that 26% of the visits made to primary care centres were prevention-related [48]. This ratio was reported to be 13% in another study, performed in the same province in 2013, almost 2 years after the introduction of the new system [49].

Our findings indicate a strong possibility of a decrease in the provision of family planning services. Participants reported in the interviews that family planning consultations are now provided only to those who demand this service, rather than trying to reach the whole community. They also reported that intrauterine devices are not fitted any more in primary care, as also noted by Kringos et al. [18]. Data representing the situation in different provinces of Turkey are in line with our findings. Eskisehir has faced a 10.3% decrease in the use of effective methods of contraception, such as intrauterine devices, oral contraceptives, and condoms [35]. In Izmir, the percentage of use of effective methods among married women aged 15-49 years decreased from 62% to 38% between 2006 and 2010; a number of problems in the provision of education and counselling services were observed in the Ministry of Health audits [50].

Atun et al. [29], who explored services provided by primary care physicians before and after the introduction of the family medicine model, observed a remarkable decrease in mobile service availability for antenatal care. Many of our participants reported that community-based activities, which were performed mainly by midwives with the primary objective of promoting the health of mothers and children while considering the social characteristics of the population, came to a halt after the introduction of the family medicine model. Special importance is given to community workers, who have a similar role as health centre nurses or midwives in many countries, considering their critical function in connecting primary care with the general public [51]. Kringos et al. reported that FPs were not the first contact of choice for sexual, psychiatric, or relationship problems and many patients were also not sure if their FP would be the right person to approach to discuss non-medical problems that impacted health [18]. These findings support our claim that physicians are not the most appropriate provider in building this connection with the public. Therefore, the abolition of the community-based activities of midwives and nurses is a very serious lost opportunity regarding the social dimension of accessibility. Lionis et al. reported a similar situation in Greece and stated that the absence of preventive and health-promotion services in the community, as well as the fact that the role of community nurses and social workers is being undervalued, seem to contribute to the low level of integrated primary care in Greece [17].

Coordination is the fourth cardinal function of primary care [2]. Kringos et al. [18] reported lack of coordination of a care as a major problem for the two pilot provinces. The authors stated that lack of multidisciplinary team work and weak cooperation between team members in primary care are among the reasons for this problem; they noted that they found no mechanisms to promote coordination between primary and secondary care levels. Based on our findings, it appears that after the introduction of the family medicine model at the national level, coordination continues to be a major problem. According to participants’ statements, this situation is caused by all three sides. For various reasons, such as the high number of patients at their daily clinics, FPs do not question their patients enough about services they have received elsewhere; the majority of patients regard giving information to their FPs or even visiting their FPs as unnecessary; and specialists do not feel it is necessary to make use of the information coming from primary care providers. Another reason is the lack of mechanisms, defined by Starfield [2], such as gatekeeping, payment incentives, official instructions, and electronic medical records. On the other hand, FPs reported that some patients with a high level of education and health awareness informed them of the services they had received elsewhere; for these patients they had been able to ensure at least partial coordination. Also, O’Malley reported a strong relationship between the level of coordination and the extent to which patients took an active role in their own care [52]. However, it should be noted that achieving coordination only for patients who are more educated and aware will serve to increase inequalities.

Davies reported that coordination with other parts of primary care can be more difficult than coordination with specialist services [53]. This may be related to the dilution of primary care caused by separating its organisation from public health, as noted by Meads [10]. This dilution of primary care can be seen in Turkey as a consequence of the separation of the services at the individual and community levels into two different organisations, the FHCs and CHCs. In addition, limiting the responsibilities of FPs and FHWs to only patients on their own lists prevents internal coordination of primary care. Jobs which were done by a multidisciplinary team in the past are now done by two people, an FP and an FHW, i.e., family health units for only their own patients. However, it is not possible to ensure cooperation and communication between these units. In fact, it was reported that even these two-person units are internally divided along the lines of treatment services and preventive services. Whereas participants reported that there are problems with communication and cooperation even within the same FHC, they also reported that there is absolutely no collaboration between different FHCs. The end result is that it is becoming more difficult to monitor the health of the community and to deal with problems that need to be tackled at the community level, especially communicable diseases and school health. Primary care reforms have had similar consequences in other countries, such as Sweden and Bulgaria [9,12]. For instance, in Sweden, introduction of the person-list system weakened physicians’ ties to geographical areas, which implied looser ties to other members of the primary care team [9].

The core functions of primary care make it the starting point in integrating care within a health system. The conceptual framework proposed by Valentjin et al. explains the close link between primary care and the micro, meso, and macro levels of integration in health care [4]. The micro level refers to clinical integration, which defines coherence in the primary process of care delivery to individual patients and is based on a person-focused perspective and not solely on a particular condition [4,54]. Our findings reflect the fact that because the community does not use FPs as a first point of contact or as a continuous provider of care, and as coordination with care providers at other levels cannot be achieved, services are provided based on a particular condition. The meso level involves organisational and professional integration [4,54]. Organisational integration is based on the principle that the needs of a population require collective action of organisations across the entire care continuum, whereas professional integration refers to partnerships between professionals both within and between organisations [4,54,55]. According to participants’ statements, cooperation with social services institutions is practically non-existent and there are important collective action deficiencies between family health units within FHCs, between different FHCs, between FHCs and CHCs, and between primary care providers and providers at other levels. Kringos et al. [18], in considering the percentage of FPs having regular meetings with local authorities, social workers, or religious groups, also criticised the weak links with the community.

Macro-level system integration encapsulates a holistic approach that puts the health needs of a population at the heart of the system to meet the needs of the population [4,55]. Therefore, the macro level can be considered as community-oriented primary care concerned with the health care needs not only of the patients and families being seen by the provider, but also of people in the community whose health care needs are not being met; it also involves characteristics of communities (including political, economic, social, and environmental) that influence the health care needs of everyone in the community [2,4]. Plochg and Klazinga [56] emphasised that community-based integrated care promotes integration of public health functions, medical care functions, and social services on a local or regional level. From this perspective, the family medicine model as applied in Turkey, which has abolished the geographically organised system and separated primary care from public health, lacks the mechanisms required to consider political, economic, social, and environmental factors. Moreover, it is concerned only with the patients being seen by FPs. Therefore, on the macro level of integration the family medicine model cannot be seen as a success.

## Conclusions

Analysis of the statements of primary care workers who participated in this study point to the family medicine model in Turkey being far from achieving the cardinal functions of primary care. This picture of primary care should be considered as a result of reforms that have mainly two components. The first component, which can be summarised as a move from a community-based approach to professional-based primary care, has led to problems such as a separation of primary care from public health, a lack of teamwork, and erosion in the perception of responsibility for the whole community. The second component is related to the introduction of incentive-payment schemes and constitutes the main underlying reason of problems regarding the discriminatory patient-selection practices of FPs, “supplier-reduced” demand through the decline of consultation time, and underprovision of services not included in the list of target payments. Because of all these facts, the family medicine model in Turkey is unable to provide integration even within itself, let alone with community health services, specialist services, or social services.

## Competing interests

The authors declare that they have no competing interests.

## Authors’ contributions

ZÖ contributed to the conception and design of the study, obtained ethical approval, conducted the interviews and interpreted the data. ZÖ and MÇ both coded the interview transcripts and discussed the codes as well as the emerging discourses. ZÖ drafted the manuscript, which was extensively commented on by MÇ and UY. MÇ participated in the design of the study, the development of a conceptual framework. UY was involved in the design of the study, the collection of data, and the revision of the manuscript and RÖ conducted the interviews, participated in the coordination of the study and provided support and critical analysis of the manuscript. All authors critically revised and approved the final manuscript.

## Pre-publication history

The pre-publication history for this paper can be accessed here:

http://www.biomedcentral.com/1471-2296/15/38/prepub
