# Erratum: Family medicine model in Turkey: a qualitative assessment from the perspectives of primary care workers

**DOI:** 10.1186/s12875-015-0293-y

**Published:** 2015-06-26

**Authors:** Zeliha Asli Öcek, Meltem Çiçeklioğlu, Ummahan Yücel, Raziye Özdemir

**Affiliations:** Ege University Faculty of Medicine, Department of Public Health, 35100 Izmir, Turkey; Ege University Izmir Atatürk School of Health, Department of Midwifery, Izmir, Turkey; Karabuk University School of Health, Department of Midwifery, Karabuk, Turkey

## Erratum

We acknowledge that in the published work [1], we presented some of the data that was previously published by the Turkish Medical Association as a report in Turkish language [2] and inadvertently failed to include this paper in the reference list. We would like to clarify that the Turkish Medical Association has allowed us to reuse the data, tables and figures presented in that reports under Creative Commons Attribution License. Additionally, we would like to clarify some of the discrepancies between the BMC Family Practice manuscript [1] and our previous report [2]. The first point is the difference in the number of questions represented in the report (14 questions) and in the article (8 questions). The report [2] is on a comprehensive project and in addition to all structural and functional elements of family medicine model it includes data on the working conditions of the primary care workers, whereas the content of the article [1] is limited with the core functions of the primary care. The second point is on the number of Family Health Workers. In the report [2] we have noted that 40 Family Health Workers and 4 midwives, who were working in small health setting in rural areas, participated in the study. In the article [1], considering that these midwives working in rural areas do not have any important difference from the Family Health Workers regarding their function and experiences, their number (4 midwives) was added to the number of Family Health Workers. We apologize for any inconvenience caused.

## References

[1] Öcek Z, Çiçeklioğlu M, Yücel U, Özdemir R (2014). Family medicine model in Turkey: a qualitative assessment from the perspectives of primary care workers. BMC Fam Pract.

[2] Öcek Z, Çiçeklioğlu M, Yücel U, Özdemir R, Türk M, Taner Ş. How did the Family Medicine Model Transform the Primary Care Environment. (Aile Hekimliği Birinci Basamak Sağlık Ortamını Nasıl Dönüştürdü?) Ankara: Turkish Medical Association Publication; 2013 [http://www.ttb.org.tr/kutuphane/aile_hekimligi.pdf].

